# Treatment of Epilepsy

**Published:** 1853-05

**Authors:** 


*Treatment of Epilepsy.* By Horace Green, M. D., Prof. of the Theory and Practice of Medicine in the New York Medical College. (New York Medical Gazette.)

Viewing Epilepsy, when unconnected with organic lesion, to depend upon laryngismus, Dr. Green was induced to try the effects of cauterization of the larynx with the nitrate of silver, in a case which presented itself some time since.

The patient was thirty-seven years old, and had epileptic fits during a period of twenty-seven years. It was supposed, in its commencement, to depend on the presence of worms. At first the spasms occurred once in two weeks. In some instances he would escape for a month, or even two months, but ordinarily the attacks were about once a fortnight. This continued until he was nineteen years old, unrelieved, apparently, by any of the many remedies employed. At this period, the patient, at the advice of his physician, commenced the internal use of the nitrate of silver. Beginning with the eighth of a grain, twice a day, the amount was gradually increased to half a grain twice daily, and, subsequently, thrice daily, and so continued for a period of *two years.* The attacks of epilepsy diminished in frequency, until, at the end of two years from the time of commencing the use of the remedy, the disease ceased altogether. But long before the disease disappeared, the nitrate made its appearance on the skin, which has continued to deepen up to the present time through a period of fifteen years.

After two years the disease returned, gradually becoming more violent, and the attacks occurring at increasing shorter intervals. For the four years preceding September, 1853, they came on, not only daily, but generally several would occur every day, and not unfrequently as many as six or eight attacks would take place every twenty-four hours.

In reflecting on the case, in connection with the theory advanced by Marshall Hall, I recollected the fact, that spasm of the glottis occurring in my practice, in laryngeal disease, had, in many instances, yielded to the topical applications of nitrate of silver to the larynx; and I proposed to try the effects of *cauterization of the larynx.* With a sponge-probang I applied a strong solution of the nitrate of silver to the interior of the larynx. At this first application of the remedy to the larynx, I remarked the unusual insensibility of the parts to the local irritant; for although the ordinary caution, that of cauterizing the pharynx and opening the glottis, before passing the probang into the larynx, was not adopted, yet not the slightest cough or apparent irritation was induced, notwithstanding the sponge, charged with a strong solution of the salt, (45 grains to the ounce of water,) continued daily for five or six consecutive days; and from the first application the spasms ceased, and the patient *passed over a period of ten days without having a single epileptic attack.*

This intermission continued until the *tenth* day, when, after having been exposed to considerable excitement, he had a slight epileptic attack. Again were the applications of the nitrate renewed, and in order to produce as much effect as possible on the larynx, the strength of the solution was increased to eighty grains to the ounce of water, and the sponge-probang, nearly straight, being used, the applications were carried through the larynx and trachea to the bronchial bifurcation.

As in each instance before, the spasms ceased after continuing the application a few days, and passed the critical period; but as the patient approached the termination of this second *decade*, he was directed to take ten grains of quinine daily, for several days. This being done, the applications were continued, and the patient passed over the critical period, to the *nineteenth* day, without experiencing the slightest attack. But near the close of the *twentieth*, his disease again returned. There was no difficulty, however, in arresting the spasms by the topical treatment as soon as this period was passed; that is, no difficulty occurred in arresting entirely the attacks of epilepsy, for a period of from ten to twenty days, during which interval the patient appeared in excellent health, in good spirits, and in the possession of his ordinary mental powers.

As the patient has in only one instance, as yet, employed freely the anti-periodic remedy, it has been determined to enter again on its use, with the hope that by its full administration the intermittent character of the disease may be controlled, and the patient restored to permanent health.

The last number of "Braithwaite's Retrospect" contains an article "on the relation of Laryngismus to Epilepsy," by Dr. E. Watson, of Glasgow, in which the same treatment was practiced as that pursued by Dr. Green, without either physician knowing of the application of the same treatment by the other.—*Charleston Med. Jour. and Rev.*

